# Alternative models of consent in out-of-hospital transfusion trials: a CAN-PATT position statement supporting exception from prospective consent with opt-out notification

**DOI:** 10.1186/s13049-026-01635-z

**Published:** 2026-06-09

**Authors:** Michael Peddle, Adam Greene, Jan Trojanowski, Andrew W. Shih, Eddie Chang, Susan Nahirniak, Dallas Pearson, Oksana Prokopchuk-Gauk, Doug Martin, Charles Musuka, Cindy Seidl, Yulia Lin, Scott MacDonald, Lindsay Richards, Michael Farrell, Damien Miller, Melissa McGowan, Brodie Nolan

**Affiliations:** 1 Ornge, Mississauga, ON Canada; 2 Division of Emergency Medicine, Schulich School of Medicine, Western University, London, ON Canada; 3 British Columbia Emergency Health Services, Vancouver, BC Canada; 4 School of Medicine, Cardiff University, Cardiff, Wales UK; 5 Department of Pathology and Molecular Medicine, McMaster University, Hamilton General Hospital, Hamilton, ON Canada; 6 Shock Trauma Air Rescue Service, Calgary, AB Canada; 7 Faculty of Medicine, University of Alberta, Edmonton, AB Canada; 8 Alberta Precision Laboratories, Transfusion and Transplantation Medicine, Calgary, AB Canada; 9 Shock Trauma Air Rescue Service, Saskatoon, SK Canada; 10 Division of Transfusion Medicine, Department of Pathology and Laboratory Medicine, Saskatchewan Health Authority, Saskatoon, SK Canada; 11 College of Medicine, University of Saskatchewan, Saskatoon, SK Canada; 12 Shock Trauma Air Rescue Service, Winnipeg, MB Canada; 13 Department of Transfusion Medicine, Shared Health Manitoba, Winnipeg, MB Canada; 14 Precision Diagnostics and Therapeutics Program, Department of Laboratory Medicine and Pathobiology, Sunnybrook Health Sciences Centre, University of Toronto, Toronto, ON Canada; 15 Emergency Health Services LifeFlight, Halifax, Nova Scotia, Ontario Canada; 16 Nova Scotia Health Authority Provincial Blood Coordinating Team, Halifax, NS Canada; 17 Royal Canadian Medical Service, Canadian Armed Forces, Ottawa, ON Canada; 18 FIRST60, Li Ka Shing Knowledge Institute, St. Michael’s Hospital, Toronto, ON Canada; 19 Department of Emergency Medicine, St. Michael’s Hospital, Unity Health Toronto, 80 Bond Street, Toronto, ON M5B 1W8 Canada; 20 Division of Emergency Medicine, Department of Medicine, University of Toronto, Toronto, ON Canada; 21 Department of Emergency Medicine, Rady Faculty of Health Sciences, Max Rady College of Medicine, University of Manitoba, Winnipeg, MB Canada

**Keywords:** Trauma, Resuscitation, Research ethics, Consent, Clinical trials, Transfusion

## Abstract

Out-of-hospital blood transfusion (OHBT) trials in trauma face unique ethical and operational challenges. Patients are often incapacitated, time-critical interventions preclude traditional consent, and surrogate decision-makers are rarely accessible in the out-of-hospital environment. The Canadian Prehospital and Transport Transfusion Network (CAN-PATT) supports the use of alternative models of consent to enable rigorous evaluation of OHBT strategies. This position statement outlines the ethical and regulatory justification for an exception from prospective consent with opt-out notification model used in the SWiFT Canada (Study of Whole Blood in Frontline Trauma) pilot randomized controlled trial. Grounded in the *Tri-Council Policy Statement 2* (TCPS2) Article 3.8 governing research in medical emergencies, the SWiFT Canada model uses multi-modal notification and post-enrollment withdrawal options to balance feasibility with respect for autonomy. We argue this approach is essential to advance evidence-based transfusion practice in Canadian out-of-hospital care and serves as a national template for emergency research.

**Supplementary Information:**

The online version contains supplementary material available at 10.1186/s13049-026-01635-z.

## Introduction

The last decade has seen growing interest in out-of-hospital blood transfusion (OHBT) for critically ill or injured patients, driven by evidence that early administration of blood products improves survival [1–8]. However, there remains a lack of randomized controlled trial (RCT) evidence, particularly in large and sparsely populated countries like Canada where access and weather challenged geography pose unique challenges [4, 7–10]. The SWiFT Canada trial (ClinicalTrials.gov: NCT06495294) was launched to address this gap by comparing prehospital whole blood (WB) transfusion to component therapy (red blood cells and plasma) in trauma patients with life-threatening hemorrhage [11].

Conducting an RCT in this environment presents a fundamental ethical barrier: obtaining prospective informed consent is effectively impossible. Patients are almost universally incapacitated due to shock or altered mental status, the therapeutic window is measured in minutes, and surrogates are rarely present. Without alternative consent models, critically ill or injured patients are systematically excluded from research, leaving life-saving interventions such as blood transfusion unevaluated and perpetuating evidence gaps in trauma care.

This position statement outlines the Canadian Prehospital Transport and Transfusion Network’s (CAN-PATT) [12] support for an exception from prospective consent with opt-out notification model used in SWiFT Canada. We believe this approach is ethically justified under Canadian research guidelines, consistent with international best practice, and essential for advancing OHBT science.

### Canadian ethical regulatory framework

The *Tri-Council Policy Statement: Ethical Conduct for Research Involving Humans* (TCPS2) provides the framework for ethical guidelines for research involving human participants in Canada [13]. According to the TCPS2, traditional informed consent is an ongoing process that requires researchers to ensure potential participants are fully informed about the purpose, methods, risks, potential benefits, and alternatives of a study, as well as how their personal information will be used and their right to withdraw at any time without penalty. In the context of clinical trials, it requires the individual to have decision-making capacity at the time of enrollment and emphasizes that the researcher must confirm understanding, answer questions, and provide sufficient time for consideration before agreement is obtained.

To provide informed consent before randomization, a person must have decision-making capacity, be free of coercion, be informed with adequate information, and given adequate time for consideration [13]. These conditions can rarely be met with respect to major trauma patients. Severely injured patients often lack decisional capacity because of their injuries or due to effects of medications. Additionally, a full disclosure as imagined in traditional clinical trial settings is not possible within the constraints of the time window for effective treatment.

In circumstances where an individual cannot directly provide informed consent, policies permit a substitute decision maker (a family member, relative, or legal representative) to provide a surrogate or proxy consent to treatment or trial participation on behalf of the patient [13]. In the trauma setting, patients are frequently injured away from home, separated from family, and without any dependable means of identifying an appropriate substitute decision-maker. Furthermore, even if a substitute decision maker was present at the scene of injury, it would be challenging to verify their legal validity and authority within this time-constrained setting. Additionally, the severity of injuries may render an individual unidentifiable, presenting another impracticality to finding a substitute decision maker. Finally, the shock of any situation of trauma would potentially impact the ability of an available substitute decision maker to make time-sensitive decisions.

Within the TCPS2, Article 3.8 provides specific guidance for research in medical emergencies. It permits enrollment without prospective consent if six conditions are met:

1. A serious threat to the participant requires immediate intervention.
2. The research offers a realistic possibility of direct benefit compared to standard care.
3. The risk is no greater than standard care or is justified by potential benefit.
4. The participant is unconscious or lacks capacity to understand the research.
5. Third-party authorization cannot be secured in time despite diligent efforts.
6. No relevant prior directive by the participant is known to exist.

We interpret that the SWiFT Canada trial meets all six TCPS2 Article 3.8 conditions, as confirmed through research ethics board (REB) review and approval. Major traumatic hemorrhage is a life-threatening emergency; early transfusion offers realistic potential benefit compared to current practice; whole blood is comparable in risk to standard component therapy; patients are often incapacitated or unable to fully appreciate the nuances of a trial intervention; surrogates cannot be identified within the narrow treatment window; and existing directives (e.g., refusal of blood products) are respected in the study protocol and standard practice for OHBT.

In this manuscript, we use the term **“exception from prospective consent with opt-out notification”** to describe the SWiFT Canada approach. This model consists of: (1) emergency enrollment under TCPS2 Article 3.8 without prospective informed consent (i.e., an exception from consent at the point of intervention), paired with (2) a structured opt-out notification process that includes multi-modal participant notification and the opportunity to withdraw from future data collection. This approach is distinct from **deferred consent** (consent-to-continue after enrollment), **community consultation models alone** (which engage the public but do not replace individual consent processes), and a **full waiver of consent without notification** (where no prospective consent or post-enrollment notification is required). For clarity and consistency, we use this terminology throughout.

### The SWiFT Canada exception from prospective consent with opt-out notification model

SWiFT Canada employs a structured exception from prospective consent with opt-out notification framework rather than a full waiver of consent. A similar exception from prospective consent with opt-out notification model was approved and utilized for the Prospective Observational Study of Safety Threats and Adverse events in Trauma (PrO-STAT) study, which evaluated acute trauma resuscitations using trauma video review [14]. Unpublished data from the PrO-STAT study showed excellent uptake with the consent model, with no issues raised by family or substitute decision makers while enrolling over 500 patients over an 18-month period. The key elements of this framework include: (1) emergency enrollment under waiver of consent, (2) multi-modal notification process for patients and substitute decision makers, (3) withdrawal mechanisms, and (4) public transparency.

#### Emergency enrollment under waiver of consent

Patients are assessed for eligibility and randomized into the trial under emergency waiver of consent. Therefore, the treating clinicians do not have to delay initiating care of the patient or providing transfusions. We believe this is further justified given available data that shows delays to blood transfusion are associated with a lower chance of survival [15]. An in-hospital study demonstrated that every minute of delay to transfusion for massively bleeding injured patients was associated with a 5% increased odds of death [16]. SWiFT Canada also supports the active provision of information about the intervention to a conscious patient or substitute decision maker, to the extent they are able and wishing to receive the information, and ongoing vigilance by providers that the study participation does not violate a known prior directive (i.e. refusal of blood transfusion).

#### Multi-modal notification

The trial utilizes a multi-modal opt-out notification process to inform patients or substitute decision makers about the trial. There was deliberate intent to have notification at different timepoints to honor the continual aspect of consent as described in the TSCP-2. This includes:

1. *A general study notification letter accompanies the patient chart on hospital arrival*. This letter is made available at the time of arrival to hospital and provided to the patient or substitute decision maker. The letter describes the basis of the trial, how patient information will be used in the trial, and how a patient or family member can contact the researchers for more information should they have any questions. A copy of the study notification letter is included in Appendix A.
2. *A study bracelet with a URL and contact information that is placed on the patient at the scene*. This URL brings them to the study website which includes additional information about the study and how to contact the research team if they have any questions or concerns.
3. *A follow-up notification letter at approximately one-month post-event*. As part of the standard of care, all patients who receive blood transfusion by the enrolling critical care transport organization in SWiFT Canada receive a notification letter approximately one month after their initial date of transport. Similar to the general study information letter, the letter to patients will accompany this standard notification letter and describe the basis of the trial, how patient information will be used in the trial, and how a patient or family member can contact the researchers for more information should they have any questions, concerns or wish to opt-out from future data collection.

#### Withdrawal mechanisms

Participants or substitute decision-makers may withdraw from any future data collection or contact at any stage. Consistent with REB approval and TCPS2 guidance on emergency research, data collected prior to withdrawal are retained to preserve the scientific validity and safety evaluation of the intervention. Retained data are limited to the minimum dataset required for primary and safety outcomes and are stored in a de-identified format. The minimum dataset is a standardized, agreed-upon set of core information, including variables and outcome measures, that should be defined before initiation of the study. In the case of biospecimen collection (i.e. blood samples), data from previous biospecimens collected prior to withdrawal of participation are retained, however future sampling is not performed.

This approach is explicitly communicated in notification materials, including the initial and follow-up letters, to ensure transparency. Requests for withdrawal are honored prospectively; however, deletion of already-analyzed or de-identified data may not be feasible without compromising study integrity. This approach aligns with accepted practices in emergency research where immediate interventions preclude prospective consent, and where complete removal of early critical data could introduce bias and undermine safety monitoring.

#### Social acceptability and community engagement

To inform the design of the SWiFT Canada trial and the consent framework presented here, our team conducted a pan-Canadian community engagement study examining public perspectives on emergency trauma research, alternative consent models, and prehospital blood transfusion [17]. The study used a mixed-methods design combining a nationally representative online survey of 1,000 adult Canadians, fielded through an established public-opinion polling firm and weighted to Canadian census parameters, and 96 short in-situ semi-structured interviews conducted across four rural Ontario communities served by the participating critical-care transport organization.

Findings broadly supported the proposed model. Approximately 73% of respondents expressed trust in the Canadian healthcare system and 80% endorsed the necessity of clinical trials involving human participants. Acceptance of alternative consent models was more conditional: 40% considered them acceptable outright, 36% indicated that their support would depend on contextual factors (severity of trauma, perceived risk, and potential benefit), and 23% were opposed. Importantly for the present framework, a majority of respondents agreed that if consent is later withdrawn by a patient or substitute decision maker, data collected prior to withdrawal should remain usable for research purposes, and 63% supported registries that collect de-identified data without prospective consent provided the data could not be linked back to the individual. The qualitative interviews reinforced these patterns and emphasised three conditions for public acceptance: transparency, proportionality (i.e., reserving alternative consent for genuine emergencies rather than as a default), and timely follow-up communication from a trusted clinician. These conditions map directly onto the multi-modal notification and post-enrollment withdrawal mechanisms described above.

#### Public transparency

Study information is posted in receiving trauma centres in public facing areas and online to enable community awareness and opt-out requests. The study team also disseminated information about the study through attendance at community events, hosting online town halls, and promoting the site on social media. There is also a planned knowledge translation plan for the results of the study via social media, conventional media (radio, television, news outlets), and scientific conferences.

Overall, we consider this approach to respect patient autonomy in a retrospective and context-appropriate manner, while prioritizing beneficence, justice, and feasibility in emergency conditions.

### Ethical justification

The SWiFT Canada model aligns with the three core principles of Canadian research ethics. Specifically these are:

1. **Respect for Persons**: The use of a multi-modal notification and withdrawal options provide meaningful autonomy despite the emergency nature of the trials and timeliness of the interventions.
2. **Concern for Welfare**: Early transfusion provides a realistic potential benefit for patients with major trauma, while research into transfusion strategies is essential to advancing knowledge on optimal resuscitation. A key limitation of the existing literature is that most studies on OHBT involve short transport times to definitive care, which does not reflect the Canadian context. As a result, their findings may not be directly applicable to the Canadian prehospital environment.
3. **Justice**: Alternative consent models allow inclusion of critically injured patients who are otherwise excluded from research, ensuring equitable access to potential benefits and reducing selection bias.

### Exception from prospective consent with opt-out notification compared to deferred consent

Deferred consent (sometimes called *delayed consent* or *consent to continue*) is a research consent model in which participants are enrolled into a study without prior consent because of the emergency or time-sensitive nature of the intervention, with formal consent sought from the patient or their substitute decision-maker as soon as it is feasible after the intervention has been delivered.

There are challenges however with using a deferred consent process for OHBT trials. First, deferred consent assumes that it will later be feasible to approach patients or substitute decision-makers after the intervention has been delivered. In reality, many trauma patients are either incapacitated, lack available family, or may not survive to hospital discharge, which makes meaningful post-enrollment consent impractical or impossible. This introduces both ethical and methodological problems, as it risks excluding precisely those patients at highest risk who stand to benefit most from early transfusion.

Second, because enrollment and intervention occur in the out-of-hospital environment, deferred consent would be logistically unworkable. It would either require (1) hospital staff, who are not part of the study team or the critical care transport organization, to obtain consent on behalf of the investigators, raising clear conflicts of role and responsibility, or (2) require the transport organization to track patients into the hospital, creating major challenges around privacy, data access, and jurisdictional authority. Both pathways introduce substantial ethical, legal, and operational barriers that make deferred consent an impractical and less acceptable model in this context.

Third, deferred consent also risks delaying transparency and autonomy. An exception from prospective consent with opt-out notification model, by contrast, emphasizes upfront communication with the public through community consultation and study dissemination, multiple future timed notifications, and ongoing opportunities to withdraw. This approach better aligns with TCPS2 by respecting autonomy while recognizing the emergency nature of out-of-hospital trauma care, where real-time informed consent is not feasible.

Lastly, we recognize that both deferred consent and exception from prospective consent with opt-out notification may be distressing for families when they are approached after the fact, particularly if the patient has died. Learning that a loved one was enrolled in a trial without their knowledge, even if the intervention was potentially lifesaving, can undermine trust in both researchers and the healthcare system. To mitigate this, the exception from prospective consent with opt-out notification incorporates a staged and sensitive notification approach for deceased patients. Initial communication is intentionally passive, with study information made available in publicly accessible hospital locations (e.g., waiting and family areas) and websites, allowing families to access information at a time of their choosing. These materials provide a brief description of the study and clear contact information for further inquiry. A notification letter is then still sent at the 1-month period to next of kin to ensure they are aware about the trial and where further information can be obtained. Any questions regarding the study that are raised by families or next of kin are directly responded back by the study principal investigator.

However, the exception from prospective consent with opt-out notification model is more flexible to allow families to grieve and process information, and reach out to the study team for information when they feel it is most appropriate, rather than being approached by someone from the study team at a set pre-determined time that is not responsive or flexible to the substitute decision maker’s needs.

### CAN-PATT position on alternative models of consent

CAN-PATT supports the use of exception from prospective consent with opt-out notification models for OHBT RCTs provided that: (1) the criteria outlined in TCPS2 Article 3.8 are fully met, (2) a multi-modal notification strategy is implemented to maintain transparency and allow patients the opportunity to withdraw, (3) appropriate community consultation and dissemination is conducted that iterates with learnings and engagement, and (4) patient withdrawals are continuously monitored with a mechanism in place to reassess the consent model if an unexpectedly high number of patients choose to opt-out. We further recommend this exception from prospective consent with opt-out notification model used in SWiFT Canada as a national template for emergency out-of-hospital research in Canada, including transfusion, resuscitation, and other time-critical interventions.

### Limitations and safeguards

This model was developed within the Canadian regulatory and geographic context, and applicability outside Canada will depend on the legal and data-protection framework of the receiving jurisdiction. Under the EU General Data Protection Regulation (GDPR), for example, emergency processing of health data without prospective consent typically rests on the scientific-research lawful bases in Articles 6(1)(e) and 9(2)(j) together with the Article 89 safeguards (data minimization, pseudonymization where feasible, organizational controls), rather than on consent itself. Participants retain qualified rights to object (Article 21) and to erasure (Article 17), each of which may be limited where erasure would seriously impair research objectives, and member-state derogations under Article 89(2) introduce further variability across European jurisdictions. The notification obligations of Articles 13 and 14 are also more prescriptive than the corresponding TCPS2 expectations, although Article 14(5) excuses provision of information where it would be impossible or involve disproportionate effort, a recognition that maps reasonably well onto the out-of-hospital trauma setting.

A consequence of these provisions, and of analogous rules in other jurisdictions, is that participant control over data collected at the moment of an emergency intervention is inherently bounded. The SWiFT Canada model approaches this transparently: prospective opt-out from future data collection and contact is preserved at every stage, but retrospective erasure of already-collected, aggregated, or de-identified data is not guaranteed where deletion would compromise scientific validity or safety monitoring. This limit is consistent with research-specific carve-outs in both TCPS2 and the GDPR, but should be made explicit and operationalised through clear notification language and pre-specified retention rules in any jurisdiction adapting this approach. Investigators outside Canada should also consider parallel instruments such as the US FDA Exception From Informed Consent regulations (21 CFR 50.24), the Declaration of Helsinki, and the CIOMS International Ethical Guidelines, which converge on similar ethical principles but differ in operational requirements like community consultation and public disclosure.

Key indicators of concern include unexpectedly high opt-out rates, recurrent participant or family concerns, or evidence of misunderstanding of study participation. These signals should prompt reassessment of the consent model and associated processes. Importantly, implementation must remain REB-specific and responsive to local populations, including Indigenous and rural communities, where expectations for engagement and governance may differ.

## Conclusion

Out-of-hospital transfusion trials are essential to guide evidence-based practice and blood system policy in Canada. Without alternative consent models, these trials are not feasible and unjustly preclude important research to improve care of major trauma patients. The SWiFT Canada exception from prospective consent with opt-out notification framework balances feasibility with respect for autonomy and meets the required ethical standards under TCPS2. CAN-PATT endorses this model as a necessary and appropriate approach to enable research that will ultimately improve survival for Canadians with life-threatening hemorrhage requiring management prior to hospital arrival.

## Electronic supplementary material

Below is the link to the electronic supplementary material.

Supplementary Material 1: Appendix. SWiFT Canada notification of study letter.

## Data Availability

Data sharing is not applicable to this article as no datasets were generated or analysed during the current study.

## Abbreviations

CAN-PATT: Canadian Prehospital Transport and Transfusion Network
CIOMS: Council for International Organizations of Medical Sciences
FDA: Food and Drug Administration
GDPR: General Data Protection Regulation
OHBT: Out-of-hospital blood transfusion
PrO-STAT: Prospective Observational Study of Safety Threats and Adverse events in Trauma
RCT: Randomized controlled trial
REB: Research ethics board
SWiFT Canada: Study of Whole blood in Frontline Trauma Canada
TCPS2: Tri-Council Policy Statement: Ethical Conduct for Research Involving Humans
URL: Uniform resource locator
WB: Whole blood
