# Supplementary material for: Alternative models of consent in out-of-hospital transfusion trials: a CAN-PATT position statement supporting exception from prospective consent with opt-out notification
Source: Scand J Trauma Resusc Emerg Med. 2026 Jun 9;34(Suppl 1):101. doi: 10.1186/s13049-026-01635-z (PMC13250996; doi:10.1186/s13049-026-01635-z)
Supplement: Supplementary file 1 — Supplementary Material 1: Appendix. SWiFT Canada notification of study letter. [file 13049_2026_1635_MOESM1_ESM.docx]

**STUDY NOTIFICATION**


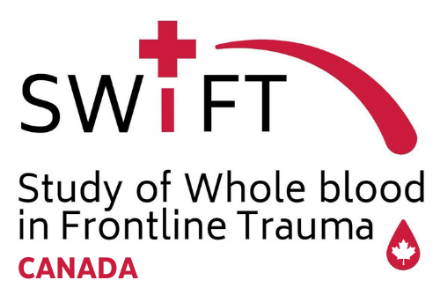


**Study Title: SWiFT Canada** (Study of Whole Blood in Frontline Trauma): A Pilot Randomized Controlled Trial Assessing Prehospital Whole Blood versus Component Therapy in Traumatic Hemorrhage

This notification is intended for the person who is eligible to take part in this study. Please note that the use of the term “you” in this document refers to the person who is eligible to participate in this study.

A team of researchers in Ontario would like to share information about a study because you received emergency care by Ornge Air Ambulance. Thank you for taking the time to read this study notification.

**Why Are We Doing This Study?**

The purpose of this study is to examine various blood transfusion treatments administered to patients experiencing severe bleeding with major injuries, with the goal of enhancing the quality of care.

**Why Am I Receiving This Notification?**

You recently experienced severe bleeding with major injuries and required a blood transfusion as part of the prehospital emergency care provided by Ornge Air Ambulance.

While you were being treated by Ornge Air Ambulance, you were unable to clearly communicate your preferences, and you were enrolled in the SWIFT Canada study.

You were enrolled into the study by Ornge Air Ambulance paramedics who first cared for you and brought you to the hospital. The paramedics then gave you either whole blood or blood components (red blood cells and plasma) while you were being transferred to the hospital.

Normally we obtain written consent from participants or their family members prior to enrolling participants into a clinical study. However, due to the seriousness of your injuries and the immediate need for a blood transfusion, the paramedics were unable to ask your permission to participate in this study without delaying your urgent transfer to the hospital. The Unity Health Toronto Research Ethics Board has reviewed this study, which involves enrolment of participants without their individual consent.

At this time, we would like to provide you with additional details about the study and inquire if you would be willing to continue participating.

This research will help us understand how to improve the care of patients who experience severe bleeding after a major injury. Before making your decision about continuing in the study, it is important for you to understand its purpose and what participation will entail.

Please take time to read the following information carefully and discuss it with others if you wish.

Please ask us if there is anything that is not clear or if you would like more information.

**What Is The Purpose Of This Study?**

Every year, uncontrolled bleeding due to major injury accounts for more than 2 million deaths worldwide. This study is examining blood transfusion treatments for patients who experience severe bleeding from major injuries before arriving at the hospital.

Timely blood transfusion is an essential part in treating severe bleeding, as delays can lower the chances of survival. Every minute of delay in administering blood to someone with severe bleeding from major injury, increases the risk of

death by 5%. Transfusion may involve the use of different blood components, such as red blood cells (important for carrying oxygen around the body), plasma (contains essential proteins to help blood clot) and platelets (small cells that are essential for blood clot formation).

In Ontario, Ornge Air Ambulance helicopters treat patients with severe bleeding from major injuries using a combination of red blood cells and plasma, which are packaged in separate bags. Platelets are stored differently to other blood products and are more challenging to transport on air ambulances, so are typically administered after the patient reaches the hospital.

However, transporting separate bags containing different blood components presents several logistical challenges. This includes increased weight that the air ambulance team must manage, increased complexity as multiple bags may be required per patient, and the potential for delays in transporting a patient to hospital.

Whole blood contains red cells, plasma and platelets all in one bag, as taken from a blood donor and offers a potential solution to these challenges. Administering a blood transfusion of all components from a single bag could streamline logistics and improve patient care in these critical situations.

**Are Both Transfusion Treatments Frequently Used?**

Yes, several places in the world use both treatments (whole blood or red blood cells and plasma) to care for patients before they get to hospital. Both treatments are considered safe and whole blood is standard treatment in many areas and cities, including in North America.

In Canada, a 2023 study found that transfusion treatment varies across the country and the six critical care transport organizations (air ambulance) with a prehospital transfusion program. All are carrying at least 2 bags of red blood cells, two services also carry plasma and none currently carry platelets.

Patients who are actively bleeding are losing ‘whole blood’ and quickly replacing all components lost with whole blood transfusion treatment could improve survival. It is for these reasons that in Canada, as in many countries, there is now increasing interest in providing whole blood transfusion treatment for severe bleeding outside of hospitals, as whole blood contains platelets in addition to red blood cells and plasma.

**What Will Happen During This Study?**

The study will compare the effects of two treatments (whole blood or red blood cells and plasma) by examining survival rates and the amount of blood required during the first 24 hours after injury.

The two treatments have been randomly allocated to the Ornge Air Ambulance helicopter that came to care for you.

The Sunnybrook Health Sciences Centre Transfusion Medicine lab supplies the blood used by Ornge Air Ambulance. The lab uses a computer to randomly assign each cooler box to carry either whole blood or red blood cells and plasma. The sealed cooler boxes are then delivered to the Ornge Air Ambulance base. When Ornge Air Ambulance is called to care for a patient, the paramedics take one cooler box with them but they don’t know what treatment is in the cooler box until it is opened.

You had an equal chance 50/50 of receiving whole blood or red blood cells and plasma. Both interventions are considered standard of care at Ornge for the management of bleeding from trauma.

We are collecting health data on your progress until hospital discharge. No additional information will be asked of you.

**What Are The Possible Risks And Benefits?**

There are no known risks linked to/attributed to taking part in this study, and there are no known additional risks in participating in the study compared to the risk associated with transfusing blood components. Both treatments are

safe and widely used in North America for severe bleeding with major injuries. The treatments in this study are not investigational.

Information collected as part of your participation may benefit severe bleeding injured patients in the future.

**What Will Happen If I Do Not Want To Continue Participating In The Study?**

You have the option to continue participating in this study. If you decide to proceed, we will continue collecting information from your hospital records. No further action is required from you if you choose to continue.

If we do not receive a response from you, we will automatically use all the information we have collected for research purposes, including routine clinical information from Ornge Air Ambulance and your hospital stay.

**You may withdraw from this study up to one month after this letter is sent**. Withdrawing will not affect the care you currently receive or may receive in the future.

If you choose to withdraw, we will retain the study data collected up to the point of your withdrawal.

**Privacy and Confidentiality of Your Personally Identifying Information and Study Data**

This section describes how your personally identifying information and study data will be accessed, disclosed, and stored during this study. All persons involved in this study are committed to respecting your privacy. Other than the individuals or groups described in this section, no persons will have access to your personally identifying information without your consent, unless required by law.

Personally identifying information is any information that could be used to identify you; this includes your name and health chart number.

Study data is information that is generated by and/or collected for a study that has been stripped of personally identifying information.

**PROTECTING YOUR PRIVACY**

The study team will make every effort to keep your personally identifying information private and confidential in accordance with all applicable privacy legislation, including the Personal Health Information Protection Act (PHIPA) of Ontario.

In addition to the study team, other authorized employees of Unity Health Toronto may have access to your personally identifying information so that they can carry out regulatory or institutionally required duties.

Unity Health Toronto may also store personally identifying information that is collected or used for these duties for a period of time, in accordance with regulations and institutional policies.

No personally identifying information will be allowed off site in any form, unless required by law or as described in this notification letter.

All data collected for research purposes will be labelled with a unique study identification number instead of any of your personally identifying information. The principal investigator is in control of the key that links your study identification number to you personally and will keep it stored separately from the study data.

**MEDICAL RECORDS**

You are authorizing access to your medical records by the study team to collect information to conduct this study. We will review your medical records from both Ornge Air Ambulance and/or the hospital that cared for you. We will

collect information such as age, sex, your medical condition and the care received. This study will only use existing information from the medical records and will not seek new information.

You are also authorizing access to your medical records by representatives of the Unity Health Toronto Research Ethics Board, the study sponsor. Such access will only be used to verify the authenticity and accuracy of the information collected for this study, without violating your confidentiality, to the extent permitted by applicable laws and regulations.

**Storage and Retention of Your Study Data**

Study data will be securely stored at Unity Health Toronto. Study data may also be transferred outside of Unity Health Toronto and shared with others for purposes related to the conduct of this study.

Study data may be kept indefinitely and may be used for other research or analyses by the study investigators and the study sponsor.

Individual level study data (non-identifying) may also be made available to scientific journals, their reviewers, other researchers inside or outside of Unity Health Toronto, or the public.

**What Will Happen to the Results of the Study?**

Once the study is completed the results will be published in scientific and medical journals and presented at meetings. We will also provide a summary of the results on a dedicated study website which can be accessed at: www.first60.ca/current-studies/swift/. You will not be identifiable in any publications or presentations resulting from this study.

**Research Ethics Board Contact**

If you have any questions regarding your rights as a research participant, you may contact the Chair of the Unity Health Toronto Research Ethics Board at xxx-xxx-xxxx during business hours (9:00am to 5:00pm).

**Study Contact**

**If you have any questions about the study, or if you wish to withdraw from the study at any time, please contact** [**SWIFT@unityhealth.to**](mailto:SWIFT@unityhealth.to) **or call xxx-xxx-xxxx**

The study is being managed by FIRST60: Prehospital, Trauma and Resuscitation Sciences Team at Unity Health Toronto.

**To learn more about this research study, please visit**  www.first60.ca/current-studies/swift/

**Principal Investigator:** Brodie Nolan, Unity Health Toronto

**Investigative Team:** Yulia Lin, Sunnybrook Health Sciences Centre

Luis Da Luz, Sunnybrook Health Sciences Centre

Michael Peddle, Ornge Air Ambulance

Ian Drennan, Ornge Air Ambulance

Jeannie Callum, Kingston Health Sciences Centre

Andrew Beckett, Unity Health Toronto

Katerina Pavenski, Unity Health Toronto

Johnathan Mack, Canadian Blood Services

**Funder:** Veteran Affairs Canada
